# The natural plant stress elicitor *cis*-jasmone causes cultivar-dependent reduction in growth of the stink bug, *Euschistus heros* and associated changes in flavonoid concentrations in soybean, *Glycine max*

**DOI:** 10.1016/j.phytochem.2016.08.013

**Published:** 2016-11

**Authors:** José P. da Graça, Tatiana E. Ueda, Tatiani Janegitz, Simone S. Vieira, Mariana C. Salvador, Maria C.N. de Oliveira, Sonia M. Zingaretti, Stephen J. Powers, John A. Pickett, Michael A. Birkett, Clara B. Hoffmann-Campo

**Affiliations:** a Embrapa Centro Nacional de Pesquisa de Soja, Caixa Postal: 231, CEP. 86001-970, Londrina, PR, Brazil; b UNESP Universidade Estadual Paulista, FCAV, Via de Acesso Prof. Paulo Donato Castellane, s/n, CEP. 14884-900, Jaboticabal, SP, Brazil; c IAC Instituto Agronômico de Campinas, Av. Barão de Itapura, 1481, Cx. Postal: 28, CEP. 13012-970, Campinas, SP, Brazil; d UEL Universidade Estadual de Londrina, Rodovia Celso Garcia Cid, PR 445 Km 380, Caixa Postal 6001, CEP. 86051-980, Londrina, PR, Brazil; e UNAERP Universidade de Ribeirão Preto, Avenida Costábile Romano, Caixa Postal: 2201, CEP. 14096-900, Ribeirão Preto, SP, Brazil; f Biological Chemistry and Crop Protection Department, Rothamsted Research, Harpenden, Herts. AL5 2JQ, United Kingdom; g UEM Universidade Estadual de Maringá, Avenida Colombo, 5790, Jardim Universitario, CEP. 87020-900, Maringá, PR, Brazil; h Computational and Systems Biology Department, Rothamsted Research, Harpenden, Herts. AL5 2JQ, United Kingdom

**Keywords:** *Glycine max*, *Euschistus heros*, Defence, *cis*-jasmone, Flavonoid, Development

## Abstract

To test the hypothesis that the plant stress related elicitor *cis*-jasmone (cJ) provides protection in soybean pods against the seed-sucking stink bug pest, *Euschistus heros*, the growth of *E. heros* on cJ-treated pods was investigated using three soybean cultivars differing in insect susceptibility, i.e. BRS 134 (susceptible), IAC 100 (resistant) and Dowling (resistant). *E. heros* showed reduced weight gain when fed cJ-treated Dowling, whereas no effect on weight gain was observed when fed other treated cultivars. Using analysis of variance, a three factor (cultivar x treatment x time) interaction was observed with concentrations of the flavonoid glycosides daidzin and genistin, and their corresponding aglycones, daidzein and genistein. There were increases in genistein and genistin concentrations in cJ-treated Dowling at 144 and 120 h post treatment, respectively. Higher concentrations of malonyldaidzin and malonylgenistin in Dowling, compared to BRS 134 and IAC 100, were observed independently of time, the highest concentrations being observed in cJ-treated seeds. Levels of glycitin and malonylglycitin were higher in BRS 134 and IAC 100 compared to Dowling. Canonical variate analysis indicated daidzein (in the first two canonical variates) and genistein (in the first only) as important discriminatory variables. These results suggest that cJ treatment leads to an increase in the levels of potentially defensive isoflavonoids in immature soybean seeds, but the negative effect upon *E. heros* performance is cultivar-dependent.

## Highlights

• *cis*-Jasmone (cJ) affects levels of defence flavonoids in immature seeds of soybean, *Glycine max*.
• The weight gain of the seed sucking stink bug pest, *Euschistus heros*, is reduced when fed on cJ-treated cv. Dowling only.
• The negative effect of cJ treatment upon *E. heros* performance is cultivar dependent.

## Graphical abstract

*cis*-Jasmone treatment of soybean pods changes flavonoid levels and negatively affects *Euschistus heros* growth in a cultivar dependent manner.

**Figure:**
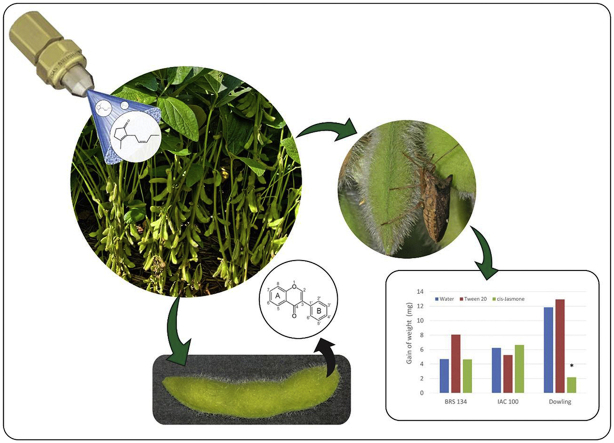

## 1 Introduction

During the last decade, soybean, *Glycine max* (L) Merrill, has been established as a major crop in Brazil. In the 2004/2005 season, Brazilian soybean production was 51 million tons (http://faostat.fao.org/site/567/default.aspx#ancor), whilst in 2015/2016, its production surpassed 100 million tons, with an average productivity of 3.037 kg/ha (http://www.conab.gov.br/OlalaCMS/uploads/arquivos/16_02_04_11_21_34_boletim_graos_fevereiro_2016_ok.pdf). Soybean yields could be potentially higher, but are affected by biotic factors including insect damage. During pod development, soybean is attacked by stink bugs (Heteroptera: Pentatomidae) that directly injure pods and seeds, causing adverse effects on seed yield (Hoffmann-Campo et al., 2003). According to Corrêa-Ferreira and Azevedo (2002), stink bug damage affects pod filling, causing severe reduction in seed vigour and viability, and increased pathogen infection.

At the beginning of the present century, populations of the Neotropical brown stink bug pest, *Euschistus heros* (Fabricius), were restricted to small areas of soybean crops in Brazil. Due to its ability to adapt to different agro-ecological zones, it has become virtually the only seed-sucking insect in soybean fields, spreading widely across the Brazilian soybean planting area (Panizzi et al., 2012). *E. heros* is now the most abundant species in Brazil, and its control is almost exclusively *via* insecticides. Furthermore, *E. heros* has now developed resistance to most insecticides recommended for its control (Sosa-Gómez et al., 2009), so alternative control strategies are urgently required, including development of pest-resistant cultivars with elevated secondary metabolite resistance traits.

For soybean, flavonoids offer protection against pests, with their production being constitutive as well as being induced by pathogen and insect damage (Dixon and Steele, 1999, Vogt, 2010). Rutin and genistin are produced constitutively in soybean PI 227697 (‘Mikayo White’) leaves, and negatively affect the performance of defoliating caterpillars, *Trichoplusia ni* (Hoffmann-Campo et al., 2001) and *Anticarsia gemmatalis* (Hoffmann-Campo et al., 2006). This germplasm accession, together with PI 229358 and PI 171451, was described by Lambert and Kilen, 1984a, Lambert and Kilen, 1984b as resistant to several lepidopteran soybean insects. Furthermore, it was observed that daidzein and genistin concentrations increased in soybean seeds of PI 227697 upon damage by *Nezara viridula* stink bugs, provoking adverse effects on insect feeding preference (Piubelli et al., 2003a). An increase in the content of isoflavones was also observed for soybean pods exposed to solar UV-B radiation by Zavala et al. (2015), who also suggested antifeedant activity of such compounds against *Piezodorus guildinii,* another soybean stink bug pest. Production of defence flavonoids in soybean is under control of PAL (phenylalanine ammonia-lyase) (Sharan et al., 1998), with the isoflavones daidzein, genistein and glycitein being subsequently synthesized *via* 7-O-glycosylation and 6″-O-malonylation, and then stored in vacuoles (Du et al., 2010).

According to Ma and Zhao (2011), the phytohormone jasmonic acid (JA) induces flavonoid production and the exogenous application of methyl jasmonate (MJ) induces activity of PAL, enabling synthesis of daidzein and genistein. Previous reports have shown that *cis*-jasmone (cJ), a compound structurally related to JA, triggers defence signalling pathways distinct from JA (Bruce et al., 2008, Matthes et al., 2010), leading to reduced aphid (sucking pest) survival and increased repellency as a consequence of elevated defence metabolite production. For example, the effects observed in wheat cultivars were increased levels of benzoxazinoids and phenolic acids (Moraes et al., 2008, Bruce et al., 2003) and, for cotton, increased levels of homoterpenes (Hegde et al., 2012).

In view of these previous observations, the performance of *E. heros* on pods of three soybean cultivars (BRS 134, IAC 100 and Dowling) that differ in levels of herbivore susceptibility was investigated following treatment with cJ. The cultivars were selected on the basis of their use in Embrapa's soybean breeding programmes, aimed at increasing tolerance and/or resistance to stink bugs (C.A.A. Arias, personal communication). IAC 100 exhibits non-preference-type (antixenosis) resistance to *E. heros* (Souza et al., 2014), and causes some biological effects on *N. viridula* (Piubelli et al., 2003b) and velvet bean caterpillar, *A. gemmatalis* (Piubelli et al., 2005). Dowling exhibits antixenosis and antibiosis effects against soybean aphids, *Aphis glycines* (Diaz-Montano et al., 2006, Hesler and Dashiell, 2011, Hill et al., 2004). A susceptible soybean cultivar, BRS 134, was included in the study to allow comparison of isoflavonoid content and *E. heros* performance across a range of susceptibility. In view of previous reports showing that insects with low weight gain exhibit low fecundity (Hadley, 1971), and with stink bug feeding mostly occurring during the 2nd to 8th days after adult emergence (Panizzi, 1995), cultivar resistance to *E. heros* following cJ treatment was assessed using a stink bug development assay that measured weight gain 7 days after plant treatment.

## 2 Results

### 2.1 Stink bug development bioassay

**Fig. 1:**
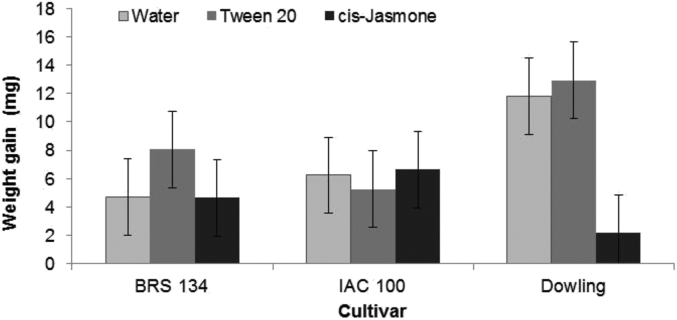
Weight gain of *Euschistus heros* fed on three soybean cultivars (BRS 134, IAC 100 and Dowling), after H_2_O, Tween 20 and *cis*-jasmone spray treatment, considering the cultivar by treatment interaction (*P* < 0.05, Table 1). Mean ± LSD (SED: 6.76) values are shown; replication, *n* = 30.

A cultivar × treatment interaction (*P* ≤ 0.05) was observed for weight gain of *E. heros* feeding on BRS 134, IAC 100 and Dowling soybean cultivars treated with water, Tween 20 and *cis*-jasmone (cJ) (Table 1). Stink bugs gained less weight when fed on cJ-treated Dowling plants than on control (water and Tween 20) treated plants. Regardless of treatment, there was no significant (*P* > 0.05) difference in the weight of *E. heros* fed on BRS 134 and IAC 100 (Fig. 1).

### 2.2 Isoflavonoid content

Results of ANOVA for concentrations of isoflavonoids in soybean seeds showed a significant interaction (*P* ≤ 0.05) between cultivar, treatment and time for daidzin, daidzein, genistin and genistein (Table 2). Contrasting variation in the concentrations of these four compounds over time was detected when plants were exposed to control and cJ treatments (see Supplemental Data). In general, genistin and genistein concentrations were higher than for other compounds, with main differences between treatments only being observed in seeds of Dowling, and only beyond the 120 h and 96 h stages respectively.

**Fig. 2:**
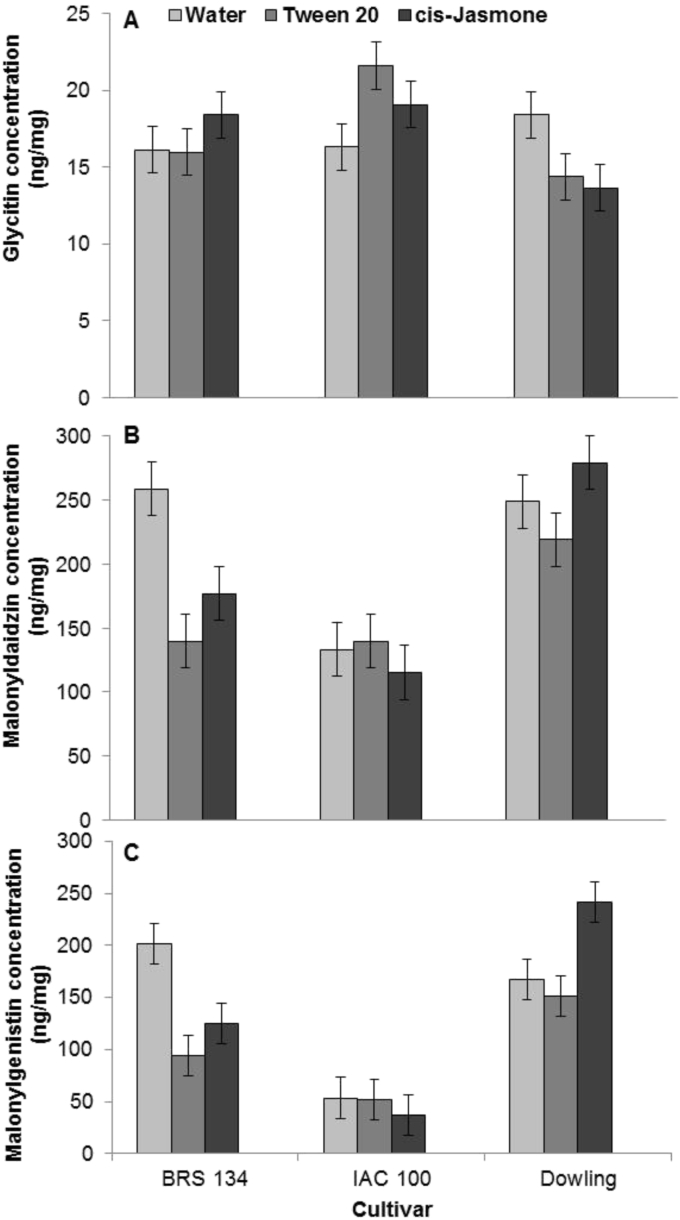
Concentrations of glycitin (A), malonyldaidzin (B) and malonylgenistin (C) in seeds removed from pods of soybean cultivars BRS 134, IAC 100 and Dowling, after H_2_O, Tween 20 and *cis*-jasmone spray treatment, considering the cultivar by treatment interaction for these isoflavonoids (*P* < 0.001, Table 2). Mean ± LSD. SED: 1.54 (Glycitin); 21.30 (Malonyldaidzin); 19.74 (Malonylgenistin), are shown; replication, *n* = 30.

For the cultivar × treatment interaction (*P* < 0.05, Table 2) for glycitin, malonyldaidzin and malonylgenistin, levels of glycitin differed in seeds of IAC 100 and Dowling between control and cJ-treated plants, but, in general, levels were 12 times lower than for the other isoflavones (Fig. 2A, B and C). In particular, levels of glycitin were lower in cJ-treated Dowling compared to the control and to levels in the other cultivars. Malonyldaidzin levels were higher in seeds of water-treated BRS 134 compared to cJ and Tween 20 treated BRS 134 (Fig. 2B). cJ-Treated Dowling had the highest levels of malonyldaidzin in comparison with the other cultivars. Levels of malonylgenistin decreased in BRS 134 treated with Tween 20 and cJ (Fig. 2C). The content of malonylgenistin in IAC 100 seeds was similar for the three treatments. However, cJ-treated Dowling plants had a higher concentration of malonylgenistin compared to controls (water and Tween 20). When comparing cultivars treated with cJ, Dowling had the highest level of malonylgenistin.

### 2.3 Canonical variate analysis

**Fig. 3:**
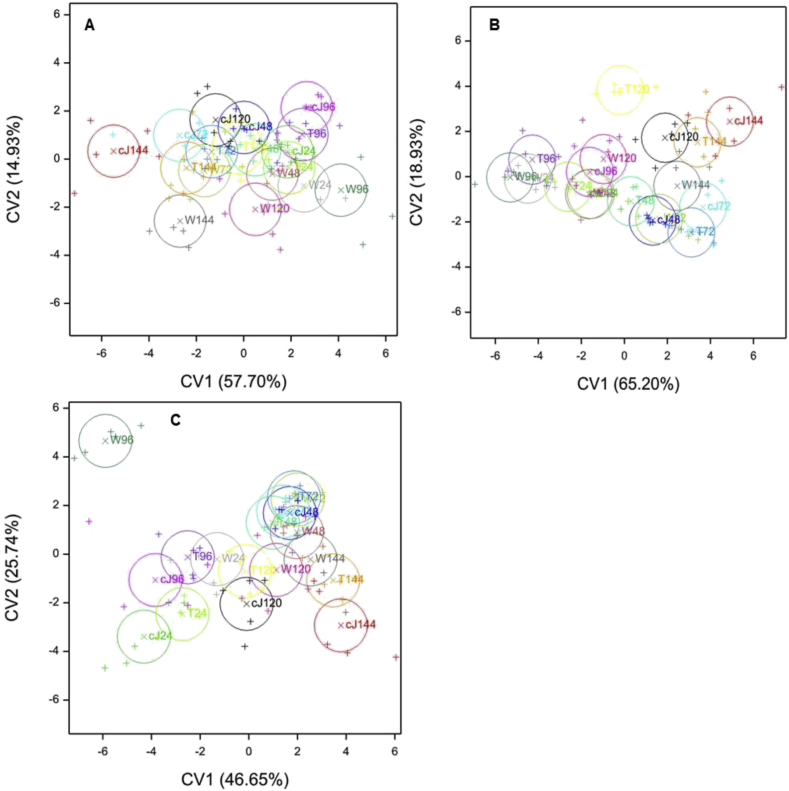
Canonical variates analysis (CVA), plots of scores (plusses) and means of scores (crosses) for combinations of treatments: H_2_O (W), Tween 20 (T), *cis*-jasmone (cJ), and time points: 24 h (24), 48 h (48), 72 h (72), 96 h (96) and 144 h (144), for each soybean cultivar BRS 134 (A), IAC 100 (B) and Dowling (C). The mean of scores (in the two dimensions, CV1 and CV2) for each combination is surrounded by a 95% confidence circle; non-overlapping circles indicate statistical difference (*P* < 0.05). The percentage variance (discrimination) accounted for in each of the dimensions is given in parenthesis with the axes labels.

Loadings from canonical variate analysis (CVA) on isoflavonoid content for each of the three soybean cultivars after spraying are shown in Table 3. For BRS 134, the first two canonical variates (CVs) accounted for 72.63% of the variation and possible discrimination between the treatments by time combinations. From the CV loadings, daidzein (CV1 and CV2), followed by genistein (CV1), were the important discriminatory variables. Hence, even though the quantities of these isoflavonoids were relatively small, they were important, as also shown by the ANOVA. The time effect was generally separated on the CV1 axis and the treatment effect on the CV2 axis, but with evidence of interaction effects (Fig. 3A). There was little difference between the treatments at the early time points, although cJ treatment was significantly different (*P* < 0.05) from the water control at 48 h. A greater difference between the treatments was observed at 96 h and 120 h when Tween 20 and cJ were both divergent from the water control, albeit not from each other, and in particular at 144 h when these two treatments were also significantly (*P* < 0.05) different. For IAC 100, the first two CVs accounted for 84.13% of the possible discrimination (Table 3). The aglycones genistein, followed by daidzein, were important discriminatory variables with respect to CV1. Daidzein was also overwhelmingly discriminatory for CV2.

The responses of cultivars to treatments became more divergent at later time points (Fig. 3B), particularly at 96 h for cJ compared to the water control, and for all three treatments at 120 h, but they were then more similar at 144 h, although with the cJ treatment still being divergent from the water control. For Dowling, the first two CVs accounted for 72.39% of the possible discrimination (Table 3). Daidzein, followed by genistein, were the important discriminatory variables with respect to CV1. Daidzein was also overwhelmingly discriminatory for CV2. For this cultivar, there was a difference for Tween 20 and cJ treatments together, compared to the water control at 24 h (Fig. 3C). It is only for this cultivar that such an early effect of the treatments was seen. At 48 h and 72 h the treatments were similar, but at 96 h, Tween 20 and cJ treatments were both different from the water control. At 120 h, the treatments were similar once again, but at 144 h the cJ treatment was different from the control.

## 3 Discussion

Treatment of three soybean cultivars (BRS 134, IAC 100 and Dowling) with the plant activator *cis*-jasmone (cJ) affected isoflavone content in soybean pods according to cultivar, treatment and time. The performance of *E. heros,* measured by insect weight gain, on the resistant cultivar Dowling was negatively affected, suggesting that the treatment effect on pest development is cultivar-dependent. Previous experiments (Piubelli et al., 2003a, Piubelli et al., 2005) provided evidence of a role for flavonoids in conferring soybean resistance to insect pests. Thus, it is hypothesized that cJ-induced changes in flavonoid production result in a cultivar-dependent reduced performance of stink bugs. Further work is required to test this hypothesis and to establish a causal effect for the role of cJ on flavonoid defence against pest attack, by testing plants with expression of genes for flavonoid biosynthesis modified by molecular genetic techniques, ie. knockout mutants or RNA-silenced plants.

Flavonoids are widely distributed in nature and exert different metabolic functions in plants, providing protection against abiotic stresses (e.g. UV radiation, metal ions) and biotic stresses (e.g. pathogens, weeds and insects), and also in facilitating pollination (Falcone Ferreyra et al., 2012). Of the twelve major known classes of flavonoids (Harborne, 1989), in soybean there is a predominance of flavonols (kaempferol, quercetin and isorhamnetin) and isoflavones (daidzein, genistein and glycitein) and their glycosides. Flavonols and isoflavones are more prominent in vegetative and reproductive soybean stages respectively (Song et al., 2014), and have been shown to play a role in protecting soybean against insects (Hoffmann-Campo et al., 2001, Hoffmann-Campo et al., 2006, Piubelli et al., 2003a, Salvador et al., 2010) and pathogens (Graham et al., 1990, Graham and Graham, 1991).

The soybean cultivar IAC 100 demonstrated tolerance to seed-sucking insects by exhibiting various mechanisms of compensation, mainly by replacing injured pods (Rosseto et al., 1995). Souza et al. (2015) tested seventeen cultivars under field conditions, including IAC 100, and based on these results, they also suggested tolerance as the most likely resistance mechanism. Despite attracting the same population of insects as the other genotypes, its yield was higher, and showed intermediate leaf retention and low levels of damaged seeds. Non-preference (antixenosis) of *E. heros* was also reported as an IAC 100 mechanism of resistance, due to large amount trichomes (Souza et al., 2014) and pod-hardness (Silva et al., 2014). This last feature negatively affected the number of probes by insects.

In the experiments herein, the weight gain of *E. heros* adults feeding on IAC 100 was not significantly reduced and was similar to the weight gain of *E. heros* feeding on the susceptible BRS 134. This difference between antixenosis and development (weight gain) assays suggests that, although IAC 100 can be avoided by *E. heros* due to morphological characteristics, in no-choice experiments, when forced to feed on it, no-direct resistance to this insect was observed. According to Michereff et al. (2015), the cultivars IAC 100 and Dowling possess a similar ability to attract sufficient numbers of beneficial parasitoids to control stink bug populations in field conditions. Thus, these two cultivars have important traits for breeding programs designed for stink bug control (Piubelli et al., 2003b, Michereff et al., 2015), with IAC 100 possessing an inducible trait for indirect (natural enemy) control, and Dowling possessing inducible traits for indirect control and direct control *via* toxic effects leading to reduced weight gain.

Time elapsed from spray treatment seems to be important when evaluating accumulation of metabolite contents in plants. Levels of genistin and genistein increased across time upon cJ treatment in all cultivars tested in this study. Increases in malonylgenistin and malonyldaidzin concentration were also observed in the seeds of Dowling, where the response to cJ-spraying was faster and stronger, suggesting that chemical induction may be a cultivar-dependent trait. Genistin and daidzin are believed to be toxic to, and negatively affect development of, insect pests such as *N. viridula* (Piubelli et al., 2003a), as well as the defoliators *T. ni* (Sharma and Norris, 1991, Hoffmann-Campo et al., 2001) and *A. gemmatalis* (Hoffmann-Campo et al., 2006). The aglycone genistein has also been classified as an antimicrobial compound with two important protective functions, i.e., acting as constitutive fitoantecipins or inducible phytoalexins (Dixon and Ferreira, 2002). In soybean, after 72 h, the aglycone genistein was detected in non-treated plants in all cultivars, but in cJ-treated Dowling plants, its concentration at 120 h was two-fold higher in relation to other treatments.

In the present study, differences between the levels of defence isoflavonoids were observed. Multivariate analysis revealed that the aglycones daidzein and genistein were the compounds responding most effectively to treatments and were considered as discriminators of treatment and cultivar. Thus, in addition to activating indirect defence in soybean leading to attraction of natural enemies of *E. heros* (Moraes et al., 2009), cJ has an additional effect on soybean defence by increasing production of compounds that provide direct defence. In our study, when the cultivar Dowling, as a soybean aphid resistant cultivar (Li et al., 2008), was treated with cJ, *E. heros* weight gain was reduced and the concentration of isoflavones, mainly genistein and derivatives, increased. This response can be explained by the feeding habits of brown stink bugs and aphids; both are hemipterans and may cause damage on soybean through phloem feeding (Bansal et al., 2013). Furthermore, according to Dhaubhadel et al. (2003), isoflavones are small molecules and their glycosylated derivatives are reasonably soluble in plant phloem. Thus, their mobility in plant certainly feasible, as these compounds are transported between different organs within plants. As an example, in one experiment carried out in our lab, cJ sprayed on soybean leaves increased the concentration of isoflavones in the plant roots.

To our knowledge, this study is the first to describe the impact of cJ upon isoflavonoid production in soybean plants. Insect-induced homoterpene biosynthesis from nerolidol and geranyllinalool is under the control of a cytochrome P450 monooxygenase CYP82G1 gene in *Arabidopsis thaliana* (Lee et al., 2010). Benzoxazinoid biosynthesis in cereals is under the control of Bx genes (Frey et al., 1995, Frey et al., 1997, Grün et al., 2005, Nomura et al., 2002, Nomura et al., 2003, Nomura et al., 2005, Nomura et al., 2007), with *bx2-bx5* encoding cytochrome P450 monooxygenases of the CYP71C subfamily. Flavonoid biosynthesis, which occurs *via* the phenylpropanoid pathway, also involves several cytochrome P450 gene products, and, based on this knowledge and our own work presented here, we hypothesize that cJ possesses a common feature in inducing cytochrome P450 genes involved in defence secondary metabolite biosynthesis. Modification of flavonoid levels in soybean suggests that genes involved in flavonoid biosynthesis are induced by cJ, but confirmation requires gene expression studies at a later stage. Most of the major enzymes and genes involved in the flavonoid pathways have been characterized.

The engineering of the flavonoid pathway for the production of compounds has been extensively used in industry (Tanaka et al., 2008). Production of novel flower colours in several plant species is the main utilization of such engineering, either by transcriptional down-regulation, by inactivating the key enzymes of the anthocyanin pathway, or by heterologous expression of key enzymes involved in anthocyanin biosynthesis (Tanaka et al., 2009, Tanaka et al., 2010). Metabolic engineering in plants has the potential to be applied to the production of secondary metabolites that affect the development and/or behaviour of insect pests, weeds and pathogens affecting crop plants, such that the production of food crops can be delivered securely with minimal seasonal inputs (fertilizers, herbicides and pesticides) (Pickett et al., 2014). This includes production of terpenes, benzoxazinoids, phenolic acids and flavonoids. Furthermore, defence gene expression in engineered crop plants can be potentially switched on by their linkage with an elicitor-responsive promoter sequence (Matthes et al., 2011; Birkett and Pickett, 2014).

In summary, the results in this study show that cJ can increase levels of flavonoids and reduce performance of the stink bug pest *E. heros*. For the future, a causal effect will be established for the role of cJ on inducing flavonoid defence against pest attack by testing plants with expression of genes for flavonoid biosynthesis modified by molecular genetic techniques. Inclusion of these defence traits in new pest-resistant cultivars through metabolic engineering associated with IPM (Integrated Pest Management) could provide new opportunities for sustainable crop protection that minimizes or removes the need for protection using broad-spectrum synthetic toxicants.

## 4 Experimental

### 4.1 Plant material

Soybean resistant cultivars IAC 100 and Dowling were selected on the basis of their known resistance to pest insects and were obtained from Embrapa's germplasm bank. The BRS 134 cultivar is a conventional Embrapa cultivar that possesses no insect resistant characteristics. Plants were cultivated in plastic pots (5 L) containing oxysoil substrate, under glasshouse conditions, at 28 °C temperature, 65% RH, and 14/10 h photoperiod (L/D). When they reached the R6 reproductive stage (Fehr et al., 1971), three different spray treatments were applied on plants as described below.

### 4.2 Spray treatment

Soybean plants at the R6 stage were exposed to exogenous application of the following treatments, as described by Moraes et al. (2009): distilled H_2_O (‘water’); distilled H_2_O + Tween 20 (0.1%) (‘Tween 20’); distilled H_2_O + Tween 20 (0.1%) + *cis*-jasmone (1.4 mmol^−1^) (cJ). cJ was purchased from Sigma-Aldrich^®^ SAFC Global (≥85% purity) and the concentration used was based on Moraes et al. (2009). Prior to spraying, cJ was mixed with Tween 20 and the homogenous mixture then dispersed in H_2_O by vigorous shaking. Plants treated with cJ were kept apart in the same glasshouse, divided by a double layer plastic wall, under the same conditions (temperature, RH and photoperiod as described above), to avoid cJ effects on control plants. After 24 h, the plants were tested in stink bug development assays and harvested for flavonoid analysis.

### 4.3 Insects

*E. heros* adults used in assays were obtained from the Embrapa Soybean stink bug mass-rearing laboratory (25 °C ± 1 °C, 70% RH and a 12:12 L:D photoperiod). From second instar to adult emergence, nymphs were fed on raw and peeled peanut (*Arachis hypogaea*), green bean pods (*Phaseolus vulgaris*), soybean seeds and *Ligustrum lucidum* fruits. At the fourth instar, the nymphs were transferred to transparent plastic boxes (25 cm × 20 cm × 20 cm), aired by screened windows on one side and on the lid. Sixty nymphs were placed in each box until they reached the adult stage. Forty-eight hours after adult emergence, insects were used in assays.

### 4.4 Stink bug development assay

Weight gain of *E. heros* on control and cJ-treated soybean at the R6 stage was assessed with the aid of an analytical balance (AG 285 Mettler Toledo, Greifensee, Langacher, Switzerland). Freshly-emerged adult insects (see above) were initially weighed and individualized into cages, made of two Petri dish bottom plates that were adapted for attachment to soybean pods (pods were not removed from the plant), and maintained in glasshouses (see above). After 144 h, cages were removed from plants and taken to the laboratory, where insects were weighed again. The weight gain of each insect was obtained by subtraction of initial weight. A randomized block experimental design was used, with treatments in a factorial scheme: three soybean cultivars (BRS 134, IAC 100 and Dowling) × three treatments (H_2_O; Tween 20; and cJ), and with 30 replicates (270 plants used in total).

### 4.5 Isoflavonoid extraction

The isoflavonoid quantification experiment was performed using a randomized block design, with treatments in a factorial scheme: three soybean cultivars (BRS 134, IAC 100, and Dowling) × three treatments (H_2_O; Tween 20; and cJ) × six sample collection times (24, 48, 72, 96, 120 and 144 h after spray treatment), and with five replicates, amounting to 270 plants. Seed pods at respective times were collected and submerged in liquid nitrogen, and kept at −80 °C until extraction and analysis of isoflavonoids. Seeds were removed from the pods, weighed (300 mg), ground with a pestle (2 ml microfuge tube), and extracted using MeOH: H_2_O (1.5 mL, 80:20) in an ultra-sonic bath for 20 min. Afterwards, each sample was centrifuged at 20800*g*, at 4 °C, for 12 min (Eppendorf 5417R Centrifuge, rotor FA-45-30-11, Hamburg, Germany), filtered through a Millipore^®^ membrane (0.45 μm), and analysed by high performance liquid chromatography (HPLC).

### 4.6 Isoflavonoid analysis and quantification

Methanolic extracts were analyzed by reversed phase HPLC (Shimadzu Corporation, model Prominence, Kyoto, Japan), using a C_18_ column, 250 mm × 4.6 mm id, particle size 5 μm. The machine was equipped with a CBM-20A controller, a SPD-20A detector, a DGU-20A5 degasser unit, LC-20AT pumps, a SIL-20A auto sampler, and a CTO-20A oven. Aliquots of 10 μl were analysed. The mobile phase was composed of two solvents: (A): 2% HOAc; and (B): MeOH + HOAc + *Milli*-Q^®^ water (18:1:1). A linear gradient system was used, starting at 75% of A and 25% of B, reaching 25% A and 75% B at 40 min and held for 5 min. At 45 min, the solvent system returned to the initial solvent mix, and was held for 5 min for column cleaning before the next injection. The solvent flow was 1 ml/min, and the detector was set at a wavelength of 260 nm. Concentrations of aglycones (daidzein, glycitein and genistein), as well as their glycosidic forms (daidzin, glycitin and genistin) and malonyl glycosides (malonyldaidzin, malonylglycitin and malonylgenistin) were determined by comparing peak areas, UV spectra and retention times of sample peaks to those of authentic standards (Sigma-Aldrich^®^). These standards were used to produce a calibration curve in the concentrations 6.25, 12.5, 25, 50 and 100 μg ml^−1^. In order to increase the confidence in peak identification, a solution containing a mix of standards (12.5 μg ml^−1^ of aglycones and 6.25 μg ml^−1^ of the glycosides and malonyl glycosides) was spiked into the samples. Pod walls were separately extracted and analysed, but as the isoflavone concentration was far smaller in pods than in seeds, the results for pods are not shown here.

### 4.7 Statistical analysis

Weight gain and isoflavonoid data were subjected to exploratory analysis using summary statistics, histograms and box-plots to consider their distribution and variance. Subsequently, data were shown to be distributed as approximately Normal and no transformation was required prior to further analysis. Analysis of variance (ANOVA) was applied using the Statistical Analysis System, version 9.2 (SAS, 2009), with means being compared using the least significant difference (LSD) test at the 5% (*P* < 0.05) level of significance, as provided by the GenStat statistical package (17th edition, ^©^ VSN International Ltd, Hemel Hempstead, UK).

A multivariate analysis, canonical variate analysis (CVA), was applied to the isoflavonoid data. The method performs a linear discrimination between treatment combinations and allows a low-dimensional representation of the differences between them, so that (in two-dimensional plots) overall significant difference can be assigned by way of non-overlapping 95% confidence circles around the means of canonical variate (CV) scores per treatment combination, making the assumption of a multivariate Normal distribution for the data. The magnitude of the CV loadings on the variables indicates the relative importance of the variables in the discrimination observed, for example, Krzanowski (2000). The CVA was considered for each cultivar separately and the results compared. The GenStat statistics package was used for this analysis.

## Figures and Tables

**Table 1:** Summary of ANOVA [degrees of freedom (DF) and F-statistics] for weight gain of *Euschistus heros* fed on pods of three soybean cultivars (BRS 134, IAC 100 and Dowling), after application of treatments (H_2_O, Tween 20 and *cis*-jasmone).

Sources of variation	DF	F-statistics
		Weight gain (mg)
Block	29	–
Cultivar (Cul)	2	2.48^ns^
Treatment (Trt)	2	3.83*
Cul × Trt	4	3.27*
Residue	232	–
Total	269	–

*P ≤ 0.05, ^ns^ not significant *P* > 0.05.

**Table 2:** Summary of ANOVA [degrees of freedom (DF) and F-statistics] for concentrations of isoflavonoids in seeds removed from pods of three soybean cultivars (BRS 134, IAC 100 and Dowling), after application of treatments: H_2_O, Tween 20 and *cis*-jasmone.

Sources of variation	DF	F-statistics							
		Daidzin (ng/mg)	Glycitin (ng/mg)	Genistin (ng/mg)	Malonyldaidzin (ng/mg)	Malonylglycitin (ng/mg)	Malonylgenistin (ng/mg)	Daidzein (ng/mg)	Genistein (ng/mg)
Block	4	–	–	–	–	–	–	–	–
Cultivar (Cul)	2	13.82***	7.8***	51.96***	46.45***	24.62***	76.71***	14.88***	34.47***
Treatment (Trt)	2	4.11*	0.09^ns^	5.77**	7.29**	0.40^ns^	7.72***	14.05***	7.59***
Time	5	40.19***	22.12***	33.03***	18.95***	11.55***	19.43***	23.64***	9.81***
Cul × Trt	4	5.14***	6.35***	3.06*	6.66***	1.99^ns^	9.97***	16.29***	2.58*
Cul × Time	10	12.08***	8.75***	8.94***	8.82***	5.81***	9.10***	12.92***	5.44***
Trt × Time	10	1.11^ns^	1.9*	1.66^ns^	0.69^ns^	1.33^ns^	1.55^ns^	2.87**	2.54**
Cul × Trt xTime	20	2.05**	1.14^ns^	2.43**	1.20^ns^	1.31^ns^	1.46^ns^	2.70***	2.97***
Residue	212	–	–	–	–	–	–	–	–
Total	269	–		–	–	–	–	–	–

*P ≤ 0.05, **P ≤ 0.01, ***P ≤ 0.001, ^ns^ not-significant *P* > 0.05.

**Table 3:** Canonical variate analysis (CVA) of the isoflavonoid content in seeds of three soybean cultivars. The CVA was calculated for each cultivar separately and the results compared (see Fig. 3). The table shows the loadings on the isoflavonoids and the percentage variation and possible discrimination between the treatment x time combinations, accounted for by the first two canonical variates (CV1 and CV2).

Cultivars	BRS 134		IAC 100		Dowling	
Isoflavonoids	CV1	CV2	CV1	CV2	CV1	CV2
Daidzein	−1.9431	7.2663	1.657	11.221	−13.183	15.439
Daidzin	−0.0792	0.0562	0.287	−0.149	0.106	−0.089
Genistein	1.1972	0.5248	−3.533	−1.103	1.455	−0.799
Genistin	0.0085	−0.0041	−0.029	0.039	0.010	0.003
Glycitin	−0.3211	−0.1355	0.070	0.089	0.447	0.506
Malonyldaidzin	0.0053	−0.0121	−0.056	0.031	0.006	−0.004
Malonylgenistin	0.0020	−0.0071	0.018	−0.019	−0.006	0.006
Malonylglycitin	0.1255	0.0803	−0.059	−0.030	−0.381	−0.303
Variation (%)	57.7	14.93	65.2	18.93	46.65	25.74
