# Supplementary material for: The natural plant stress elicitor cis-jasmone causes cultivar-dependent reduction in growth of the stink bug, Euschistus heros and associated changes in flavonoid concentrations in soybean, Glycine max
Source: Phytochemistry. 2016 Nov;131:84–91. doi: 10.1016/j.phytochem.2016.08.013 (PMC5055112; doi:10.1016/j.phytochem.2016.08.013)
Supplement: Supplementary file 1 [file mmc1.docx]

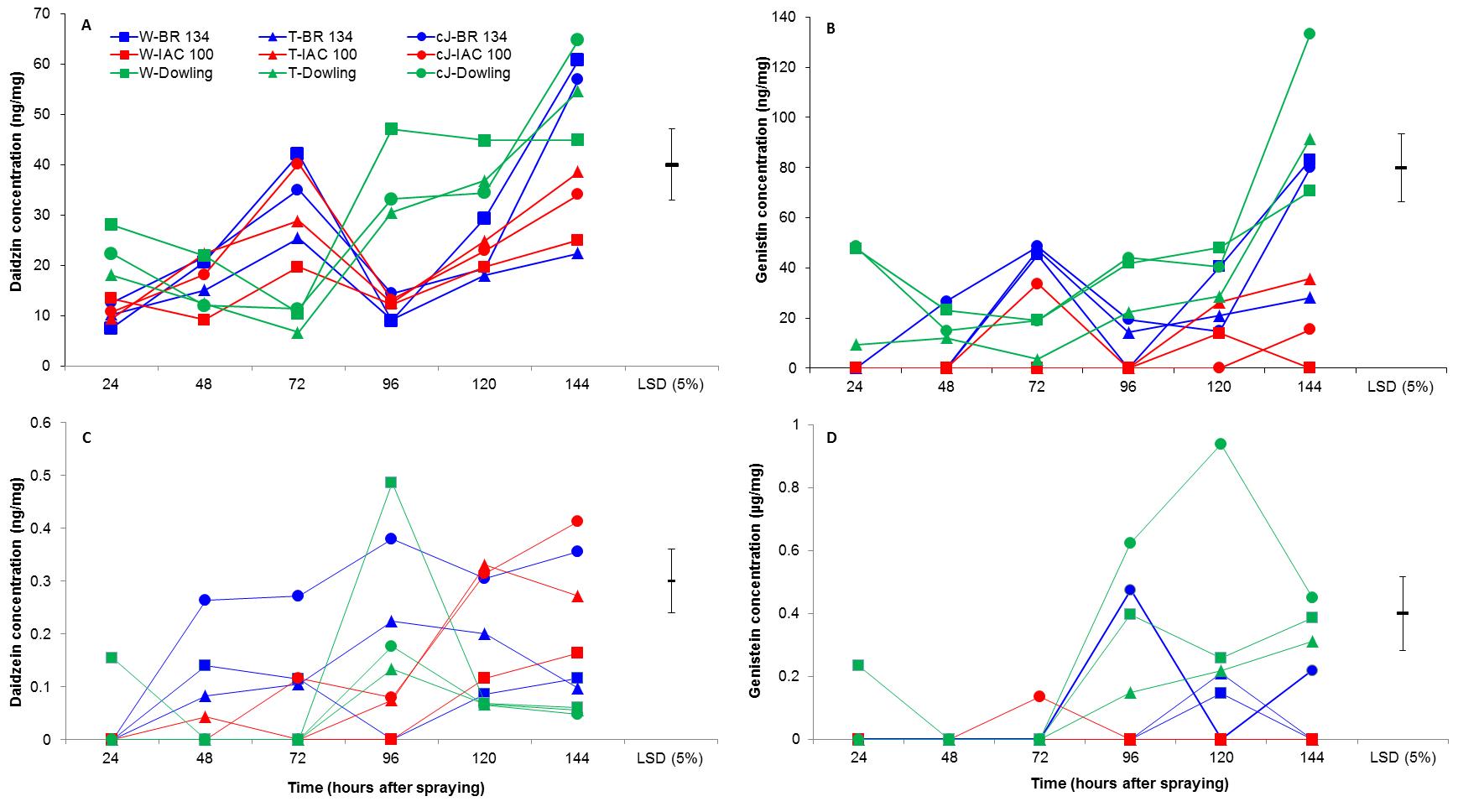


Supplementary Figure 1. Concentrations of daidzin (A), genistin (B), daidzein (C) and genistein (D) in seeds removed from pods of three soybean genotypes (BRS 134, IAC 100, and Dowling), Water, Tween 20 and *cis*-Jasmone treated. (Repetitions, *n*=5). Mean ± LSD. SED: 3.604 (Daidzin); 6.845 (Genistin); 0.303 (Daidzein); 0.0598 (Genistein).
